# Supplementary material for: A distributed and efficient population code of mixed selectivity neurons for flexible navigation decisions
Source: Nat Commun. 2023 Apr 14;14:2121. doi: 10.1038/s41467-023-37804-2 (PMC10102117; doi:10.1038/s41467-023-37804-2)
Supplement: Supplementary file 1 — Supplementary Information [file 41467_2023_37804_MOESM1_ESM.pdf]

Supplementary Information

A distributed and efficient population code of mixed selectivity neurons for flexible navigation decisions

Shinichiro Kira, Houman Safaai, Ari S. Morcos, Stefano Panzeri, Christopher D. Harvey

List of Supplementary Figures

Supplementary Fig. 1 | Behavioral performance of mice on the delayed match-to-sample task during calcium imaging experiments. . . . 2

Supplementary Fig. 2 | Photo-inhibition effects on spiking activity and the mouse's running . . . 3

Supplementary Fig. 3 | Example cell activity averaged for each task-related variable . . . 4

Supplementary Fig. 4 | GLM with various levels of explanatory power for example cells . . . 5

Supplementary Fig. 5 | Task-related information and its mixing in V1, RSC and MM . . . 6

Supplementary Fig. 6 | Task-related information in M2 and M1 . . . 8

Supplementary Fig. 7 | Population decoding with alternative methods . . . 9

Supplementary Fig. 8 | Information analysis restricted to the earlier part of the test segment (first 0.5 s) . . . 10

Supplementary Fig. 9 | Noise correlations in populations of mixed selectivity or pure selectivity cells . . . 12

Supplementary Fig. 10 | Mathematical interpretation of the decoding accuracy from simulated mixed and pure selectivity populations . . . 13

Supplementary Fig. 11 | XOR information in populations of mixed selectivity or pure selectivity cells, controlling for the population size . . . 14

Supplementary Fig. 12 | Virtual reality maze for behavioral training . . . 15

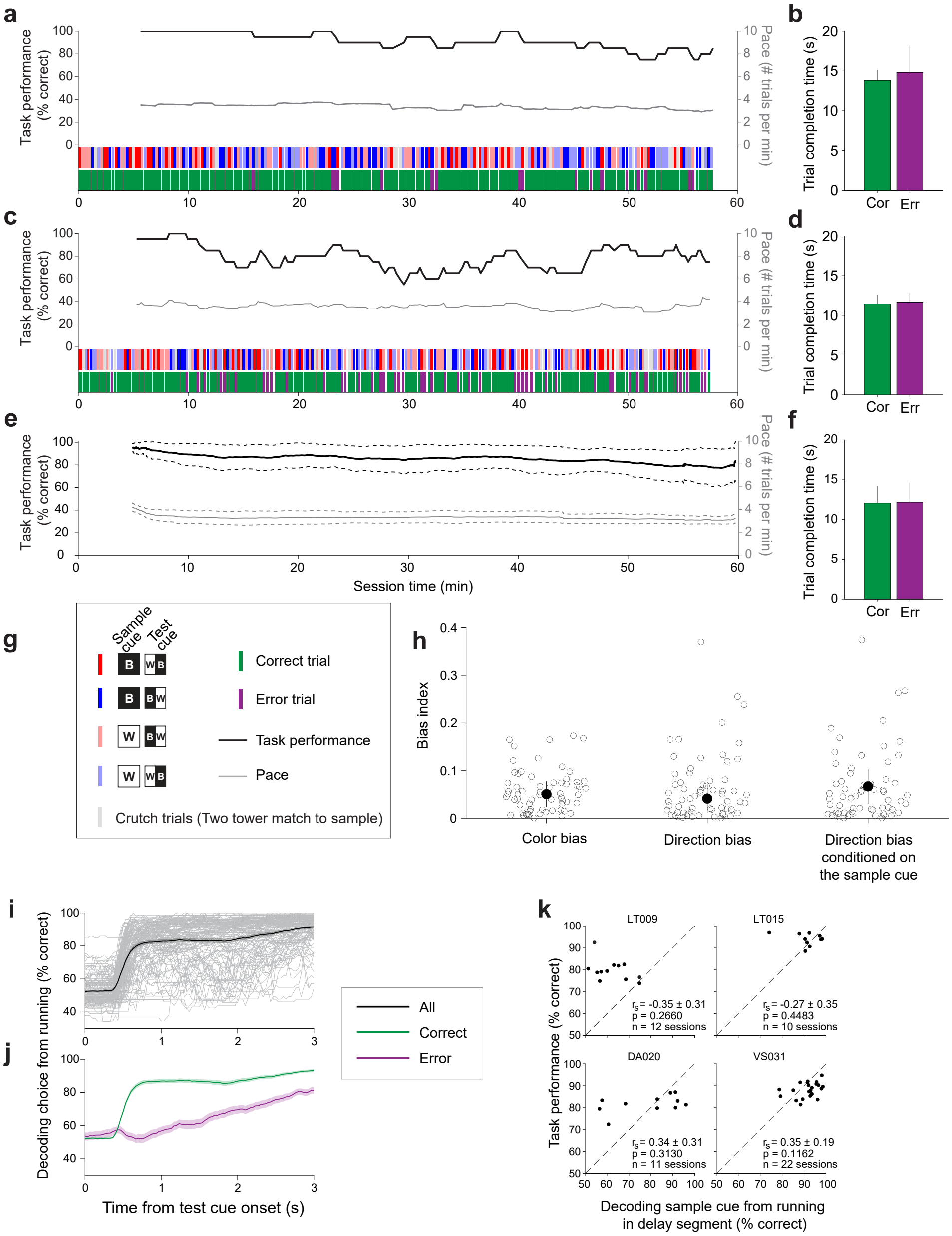

Supplementary Fig. 1 | Behavioral performance of mice on the delayed match-to-sample task during calcium imaging experiments

(a) An example session with high task performance. The raster plot shows trial types (first row) and the correctness of choice (second row). The width of rectangles and gaps between them indicate the length of trials and the inter-trial intervals, respectively. The running mean of task performance and pace were calculated from the preceding 20 trials, excluding crutch trials (see Methods). See marker legend in panel (g).

(b) Mean trial completion time for correct and error trials during the session in (a). Error bars indicate mean ± s.d. across trials. n = 179 correct / 16 error trials.

(c) Similar to panel (a), except for an example session with moderate task performance.

(d) Similar to panel (b), except for the session in (c). n = 163 correct / 44 error trials.

(e) Task performance and pace averaged across sessions. The solid and dashed lines indicate mean ± s.d. n = 63 sessions from 6 mice.

(f) Mean trial completion time for correct and error trials averaged across all sessions. Error bars indicate mean ± s.d. across sessions. n = 63 sessions from 6 mice.

(g) Marker legend for panels (a-f).

(h) Choice bias for the color (choosing black or white), direction (choosing right or left), and direction conditioned on the same sample cue (see Eq. 1-3 in Methods). Open circles show bias in individual sessions. Filled circles with error bars indicate median ± median absolute deviation. n = 63 sessions from 6 mice.

(i) Choice (final turn direction) decoded from the mouse's running velocity in the test segment. Thin gray lines indicate individual sessions. Thick black line and shading indicate mean ± s.e.m. n = 63 sessions from 6 mice.

(j) Similar to (i), plotted separately for correct and error trials. Shading indicates mean ± s.e.m.

(k) Task performance across sessions (circles) and mice (panels) compared to the decoding of the sample cue from the mouse's running velocity during the last 0.35 s in the delay segment. Two mice were excluded due to small numbers of sessions (less than five sessions). r_S indicates the Spearman rank correlation coefficient. The correlation was not significant in individual mice (p>0.11, p-values are shown for each mouse in each panel).

Source data are provided as a Source Data file.

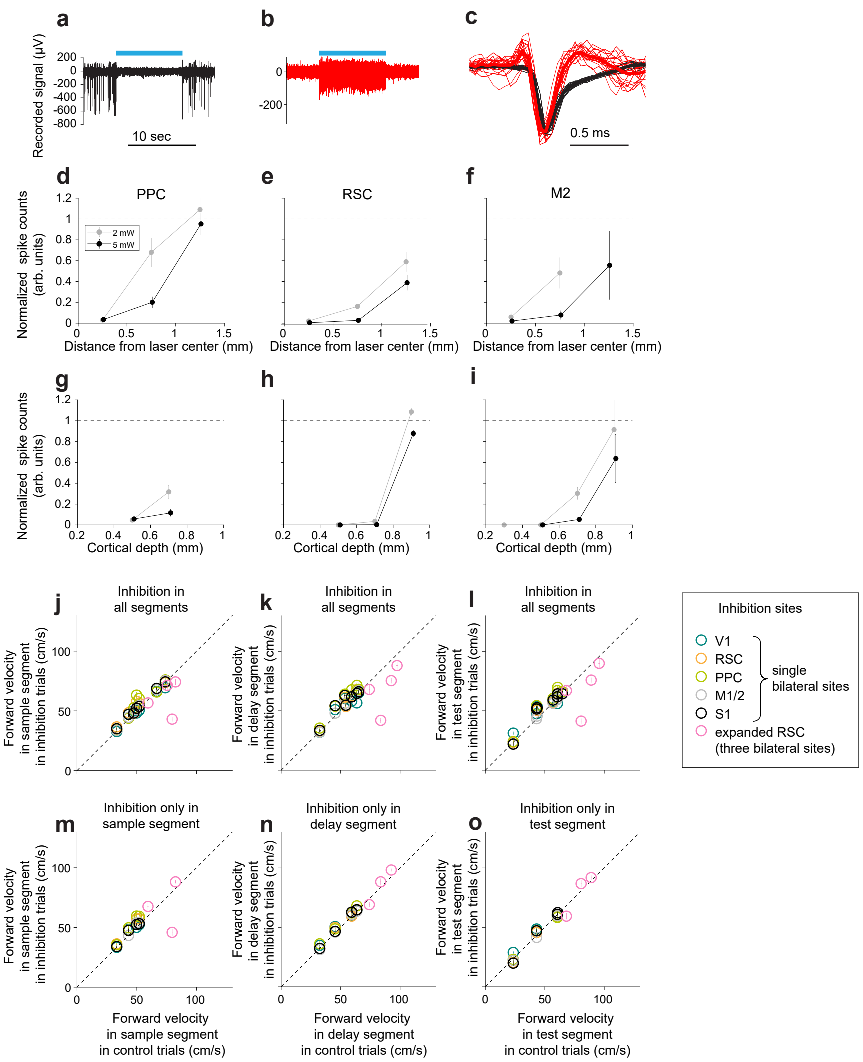

Supplementary Fig. 2 | Photo-inhibition effects on spiking activity and the mouse's running

(a) Electrophysiology trace from a putative excitatory cell in PPC of a VGAT-ChR2 mouse. Blue line indicates the photoinhibition (laser on).

(b) Similar to panel (a), except for a putative inhibitory cell.

(c) Spike waveforms for the example units shown in panels (a-b). The amplitudes were normalized to compare the spike width of a putative excitatory cell (black) and inhibitory cell (red). Thin lines indicate individual spikes. Thick lines indicate mean waveforms.

(d) Normalized spike counts (first five seconds from laser onset) as a function of horizontal distance from the laser center during photoinhibition in PPC. Spike counts were normalized to the period just before laser onset (Methods). Error bars indicate mean ± s.e.m. across trials. n = 11.8 ± 6.2 trials (mean ± s.d.) per data point from one mouse.

(e) Similar to panel (d) except for RSC.

(f) Similar to panel (d) except for M2.

(g) Normalized spike counts as a function of cortical depth from the surface in PPC. The depth was measured perpendicularly to the horizontal plane. Spikes were recorded within 500 µm horizontal distance from the laser center. n = 13.6 ± 11.2 trials (mean ± s.d.) per data point from one mouse.

(h) Similar to panel (g) except for RSC.

(i) Similar to panel (g) except for M2.

(j) Comparison of the mouse's forward running velocity in the sample segment on control trials and trials with inhibition in all segments. Each open circle indicates the average forward velocity of a single mouse on trials with inhibition of a bilateral pair of sites (or three bilateral pairs of sites for the RSC expanded inhibition) and the average forward velocity on control trials. Data from each mouse are plotted vertically at the control-trial velocity unique to that mouse. Inhibition sites are grouped for different areas as shown in Figure 1g. Error bars within open circles indicate mean ± s.e.m. across trials. n = 39 inhibition groups from 11 mice for panels (j-l)

(k) Similar to panel (j) except for the forward velocity in the delay segment.

(l) Similar to panel (j) except for the forward velocity in the test segment.

(m) Similar to panel (j) except for the forward velocity in trials with inhibition only in the sample segment. n = 23 inhibition groups from 7 mice for panels (m-o).

(n) Similar to panel (k) except for the forward velocity in trials with inhibition only in the delay segment.

(o) Similar to panel (l) except for the forward velocity in trials with inhibition only in the test segment.

Source data are provided as a Source Data file.

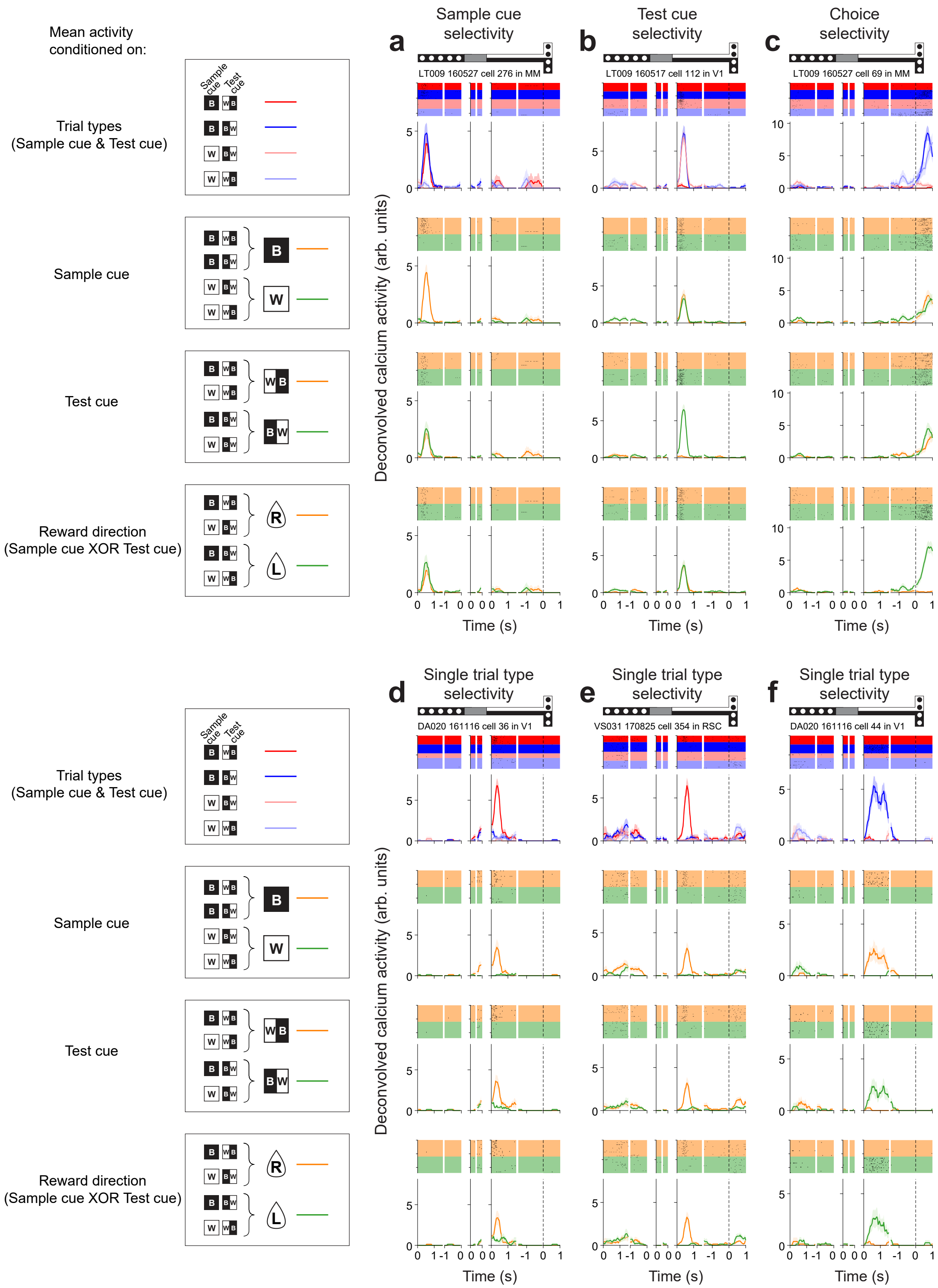

Supplementary Fig. 3 | Example cell activity averaged for each task-related variable

(a) Example cell activity with sample cue selectivity in Figure 2e. Only correct trials are included. The average activity is plotted in a different color for each trial type (1st row; same as in Fig. 2e), sample cue (2nd row), test cue (3rd row), and XOR of the sample cue and test cue (4th row). Shading indicates mean ± s.e.m.

(b) Similar to panel (a), except for the cell with test cue selectivity in Figure 2f.

(c) Similar to panel (a), except for the cell with choice selectivity in Figure 2g.

(d) Similar to panel (a), except for the cell with single-trial-type selectivity in Figure 2h. Note that the average activity of mixed selectivity cells in panels (d-f) is different for the two types of the sample cues, test cues, and XOR.

(e) Similar to panel (a), except for the cell with single-trial-type selectivity in Figure 2i.

(f) Similar to panel (a), except for the cell with single-trial-type selectivity in Figure 2j.

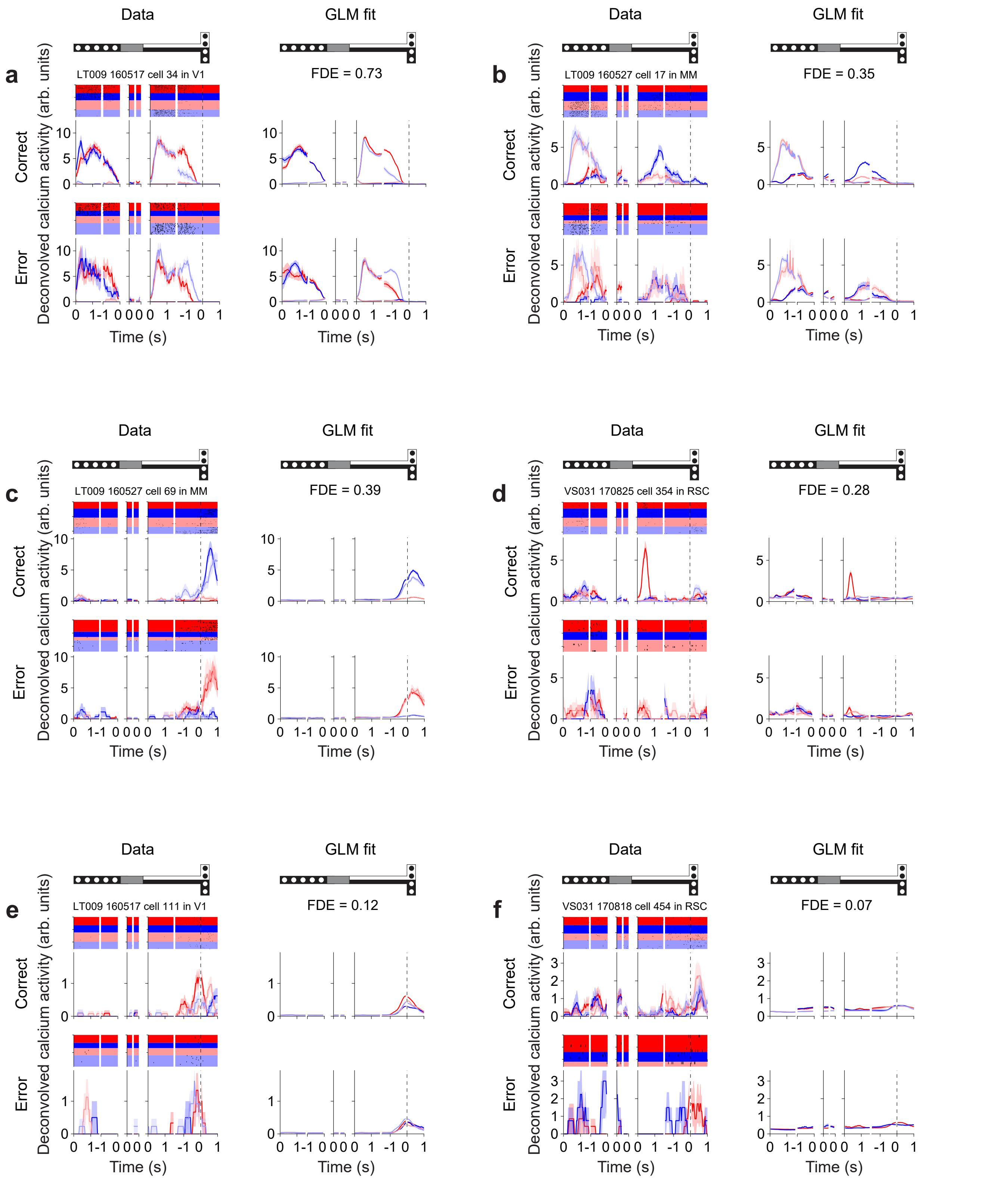

Supplementary Fig. 4 | GLM with various levels of explanatory power for example cells

(a) Observed neural activity and GLM fit for an example V1 cell. Left panels: observed neural activity plotted similarly to Figure 2e-j. Right panels: regularized GLM fits to the training data. Shading indicates mean ± s.e.m. The predictive power of the model was quantified by the fraction of deviance explained (FDE) indicated at the top.

(b) Similar to panel (a) for an example MM cell.

(c) Similar to panel (a) for an example MM cell in Figure 2g.

(d) Similar to panel (a) for an example RSC cell in Figure 2i.

(e) Similar to panel (a) for an example V1 cell with low activity and selectivity.

(f) Similar to panel (a) for an example RSC cell with low activity and selectivity.

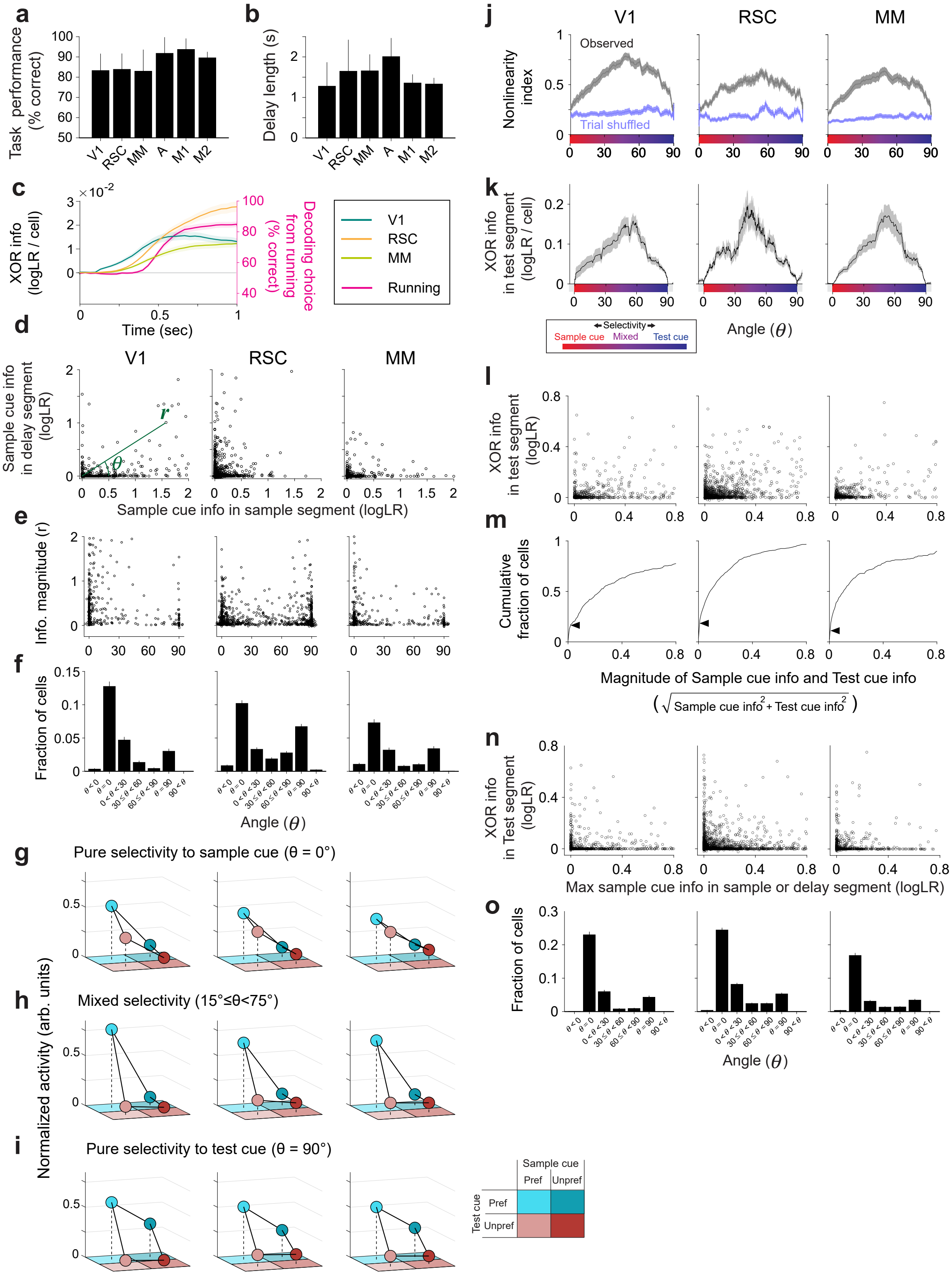

Supplementary Fig. 5 | Task-related information and its mixing in V1, RSC and MM

(a) Task performance averaged across imaging sessions for each area. Error bars indicate mean ± s.d. V1: n = 11 sessions, RSC: n = 12 sessions, MM: n = 7 sessions, A: n = 5 sessions, M1: n = 3 sessions, M2: n = 9 sessions were included in panels (a-b). Average task performance was not significantly different between two areas except for M1 and V1 (p = 0.0018) and M1 and RSC (p = 0.0011). The significance threshold was adjusted by Bonferroni correction with α = 0.05 to account for 15 between-area comparisons for panels (a-b).

(b) Delay length averaged across imaging sessions for each area. Error bars indicate mean ± s.d. Average delay length was not significantly different between two areas except for A and V1 (p = 0.0010), A and M1 (p = 0.0001), and A and M2 ($p < 10^{-4}$).

(c) Time course of XOR information and choice-related running pattern on correct trials. XOR information in V1, RSC, and MM are plotted similarly to Figure 3c. Decoding accuracy of choice in the mouse's running velocity is plotted similarly to Supplementary Figure 1j. Shading indicates mean ± s.e.m. Imaging sessions for only V1, RSC, and MM are included in the plot (n = 30 sessions from 4 mice).

(d) Sample cue information in the sample segment and delay segment for individual cells (circles) on correct trials. Cells along the horizontal axis (0 degrees) had sample cue information only in the sample segment, and cells along the vertical axis (90 degrees) had sample cue information only in the delay segment. V1: n = 1962 cells, RSC: n = 4052 cells, MM: n = 2409 cells for panels (d-f).

(e) Data from panel (d) replotted in polar coordinates as the magnitude (*r*) and angle (θ). The absence of many cells around 45 degrees indicates that few cells had sample cue information in both the sample and delay segments. Rather, different subpopulations of neurons, especially in RSC, appeared to contain sample cue information in the different segments.

(f) Distribution of cells from panel (e). Bins for $\theta = 0$ and $\theta = 90$ include cells with chance-level deviation from the axes (Methods). Error bars indicate mean ± s.e.m. Cells with noise-level information (magnitude r < 0.01) were not assigned angles but included in the total number of cells to calculate the fractions.

(g) Similar to Figure 3j. Normalized mean activity on correct trials for the four trial types for pure sample cue selectivity cells, shown separately for V1: n = 67 cells, RSC: n = 202 cells, MM: n = 54 cells. Error bars inside the colored circles indicate mean ± s.e.m.

(h) Similar to panel (g), except for mixed selectivity cells. V1: n = 142 cells, RSC: n = 383 cells, MM: n = 115 cells.

(i) Similar to panel (g), except for pure test cue selectivity cells. V1: n = 233 cells, RSC: n = 193 cells, MM: n = 123 cells.

(j) Similar to Figure 3m. Nonlinearity index on correct trials for cells across angles (running average, window of 50 cells), shown separately for V1, RSC, and MM as black traces. Cells with noise-level information (magnitude $r < 0.01$) were excluded. Blue traces show chance nonlinearity index values computed with shuffled trial identities. Shading indicates mean ± s.e.m. V1: n = 509 cells, RSC: n = 1114 cells, MM: n = 423 cells.

(k) Similar to Figure 3n. XOR information on correct trials for cells across angles (running average, window of 50 cells), shown separately for V1, RSC, and MM. Cells with noise-level information (magnitude $r < 0.01$) were excluded. Shading indicates mean ± s.e.m. V1: n = 509 cells, RSC: n = 1114 cells, MM: n = 423 cells.

(l) XOR information versus the magnitude of the sample cue and test cue information in the first one second of the test segment for individual cells (circles). Only correct trials were included in the analysis. Cells with pure XOR selectivity have high XOR information and less than the chance-level sample cue information and test cue information (Methods). For correct trials, a pure XOR selective cell or a pure choice selective cell might be active on both W/WB and B/BW trial types. Such a cell is thus active on trials with both sample cues and both test cues and lacks sample cue and test cue information (Supplementary Fig. 3c). V1: n = 1962 cells, RSC: n = 4052 cells, MM: n = 2409 cells for panels (l-m).

(m) Cumulative fraction of cells as a function of the magnitude of a cell's sample cue and test cue information in panel (l). Pure XOR selective cells were defined to have XOR information greater than logLR of 0.01 and less than the chance-level sample cue and test cue information (Methods). Cells were excluded from the analysis if they had XOR information less than logLR of 0.01. Arrow heads show the fraction of pure XOR selective cells: 16.4 ± 2.3% in V1, 18.4 ± 1.5% in RSC, and 11.1 ± 2.1% in MM (mean ± s.e.m.).

(n) Similar to panel (d), except for XOR information on correct trials in the first one second of the test segment versus the maximum sample cue information in the sample or test segment. Cells in the first quadrant ($0° < \theta < 90°$) or on the vertical axis (90°) had XOR information in the test segment with or without having sample cue information in the sample or delay segment. V1: n = 1962 cells, RSC: n = 4052 cells, MM: n = 2409 cells for panels (n-o).

(o) Similar to panel (f), except for summarizing the data in panel (n).

Source data are provided as a Source Data file.

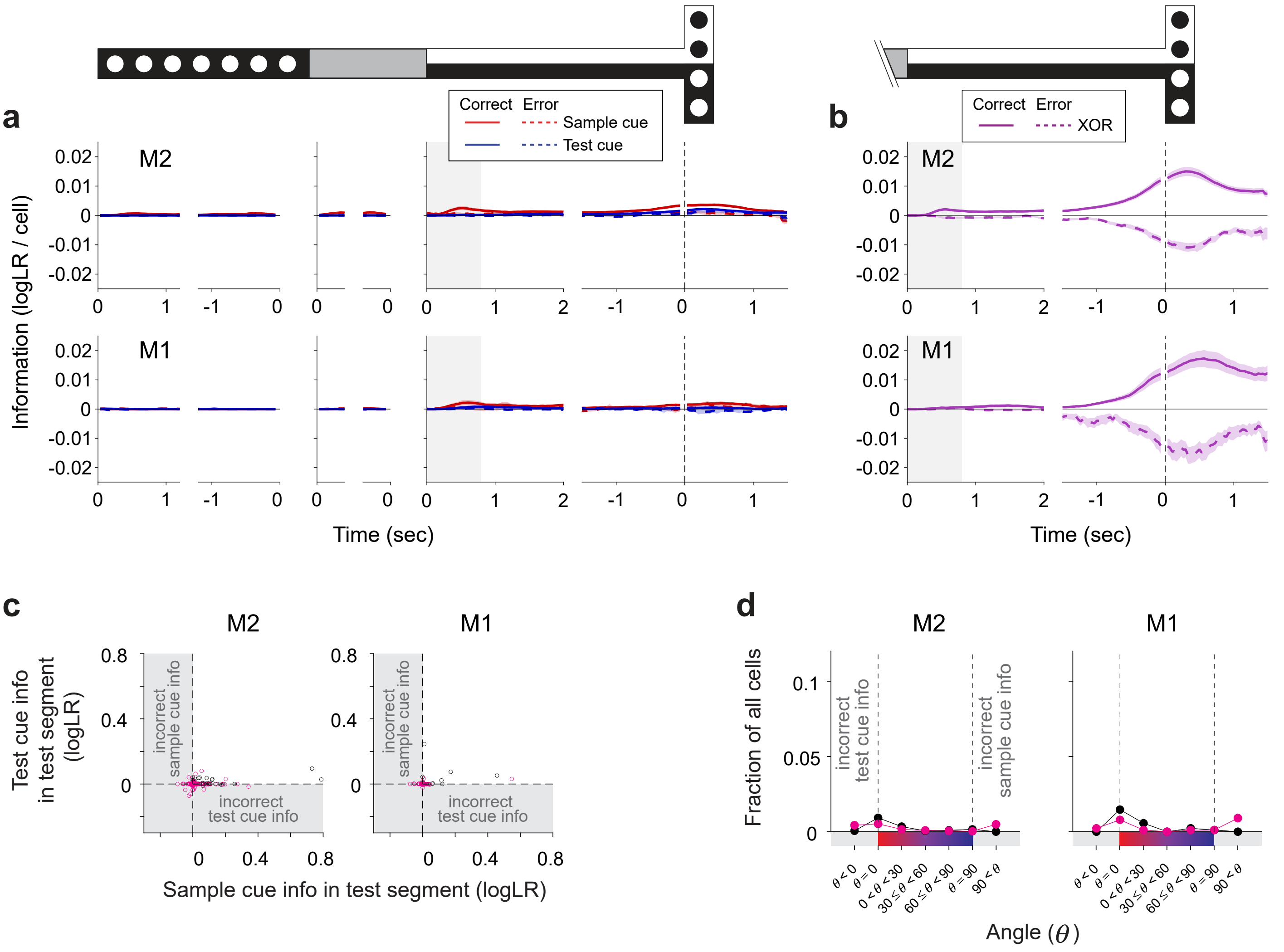

Supplementary Fig. 6 | Task-related information in M2 and M1

(a) Sample cue and test cue information quantified as logLR in individual cells in M2 and M1 for correct (solid) and error (dashed) trials, plotted similarly to Figure 4a but for a smaller range of information along the vertical axes. Shading indicates mean ± s.e.m. Gray regions indicate the period (first one second) analyzed for the test segment in panels (c-d). M2: n = 3243 cells (99.7% of detected cells), M1: n = 882 cells (99.5 %).

(b) Similar to panel (a), except for XOR information.

(c) For each cell (circle), the sample cue information in the test segment and the test cue information in the test segment on correct trials (black) and error trials (magenta), plotted similarly to Figure 4h.

(d) Distribution of cells from panel (c) in discrete polar angle bins for correct (black) and error (magenta) trials, plotted similarly to Figure 4i. Cells with noise-level information (magnitude $r < 0.01$) were not assigned angles but included in the total number of cells to calculate the fractions.

Source data are provided as a Source Data file.

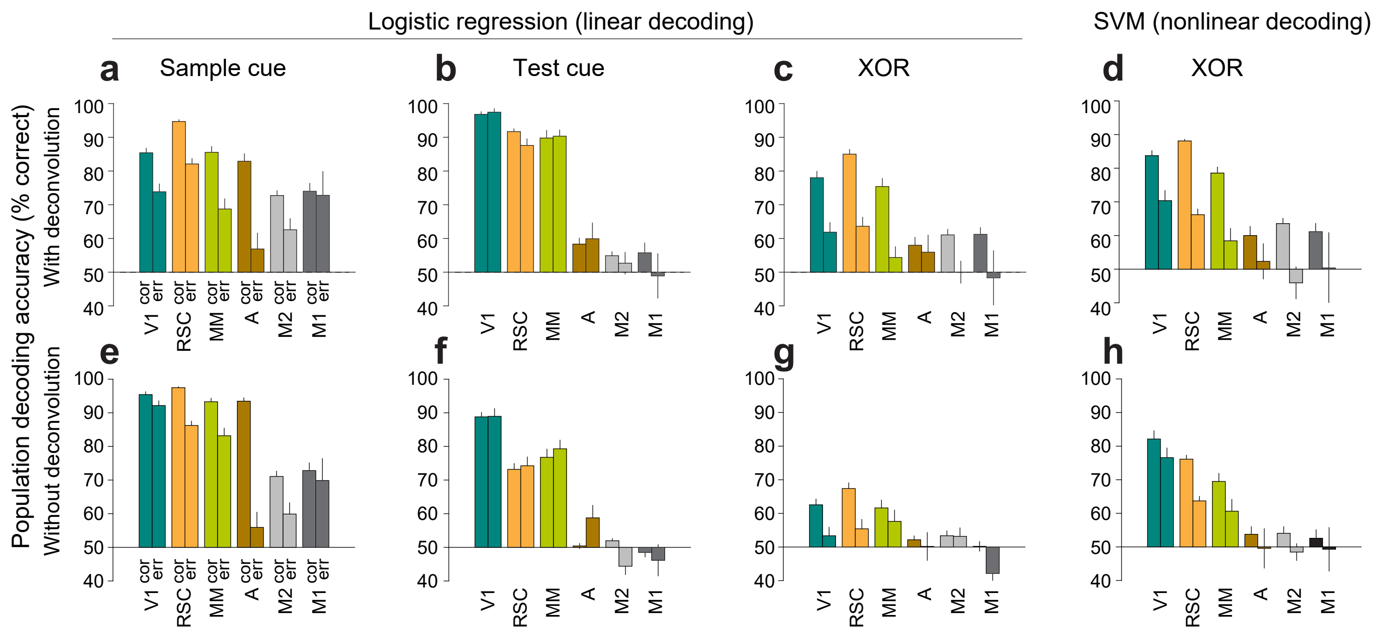

Supplementary Fig. 7 | Population decoding with alternative methods

(a) Population decoding accuracy for the sample cue identity on correct (rewarded) and error (unrewarded) trials. A linear decoder (logistic regression) was used to decode from the deconvolved activity of simultaneously imaged cells averaged across the first 1 s in the test segment (see Methods). For each session, the population activity was bootstrapped 100 times by random subsampling of 100 neurons from the entire population without replacement. Error bars show mean ± s.e.m. based on the bootstrap. V1: n = 8 sessions (1063 correct / 272 error trials), RSC: n = 12 sessions (1420 correct / 265 error trials), MM: n = 7 sessions (1010 correct / 221 error trials), A: n = 5 sessions (753 correct / 69 error trials), M2: n = 9 sessions (1313 correct / 155 error trials), M1: n = 3 sessions (458 correct / 28 error trials).

(b) Similar to panel (a), except for the test cue identity.

(c) Similar to panel (a), except for the XOR identity.

(d) Similar to panel (a), except for the XOR identity and the use of a nonlinear decoder (support vector machine with the radial basis function kernel).

(e) Similar to panel (a), except that raw dF/F was used as population activity (without deconvolution).

(f) Similar to panel (e), except for the test cue identity.

(g) Similar to panel (e), except for the XOR identity.

(h) Similar to panel (d), except that raw dF/F was used as population activity (without deconvolution).

Source data are provided as a Source Data file.

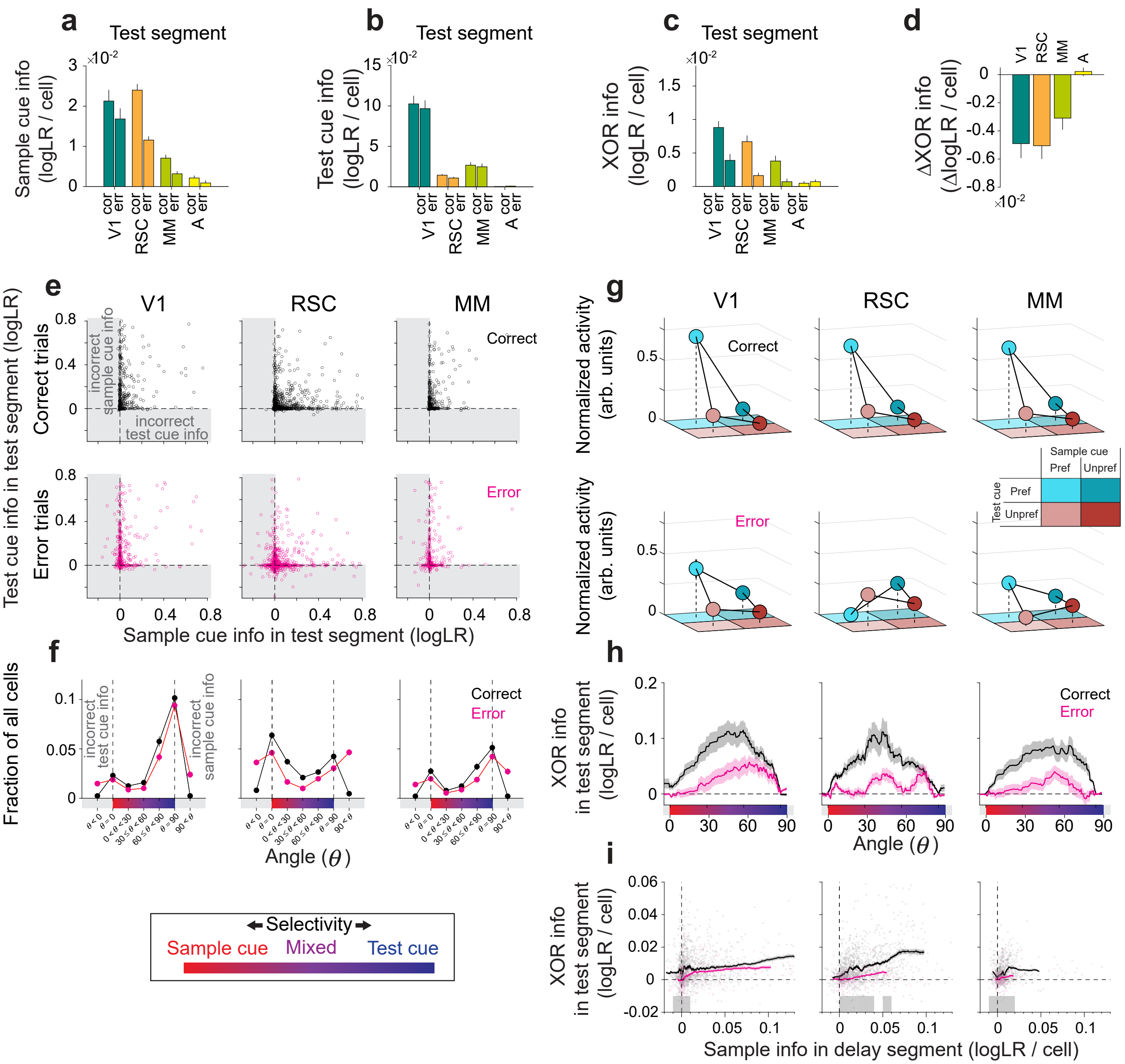

Supplementary Fig. 8 | Information analysis restricted to the earlier part of the test segment (first 0.5 s)

(a) Similar to Figure 4d, except for the first 0.5 s in the test segment. Average sample cue information per cell for correct and error trials. Error bars indicate mean ± s.e.m. The difference between correct and error trials in the sample segment was significant for RSC ($p = 0.0006$), but not for V1 ($p = 0.30$), MM ($p = 0.02$), and A ($p = 0.50$). The difference in the delay segment was significant for RSC ($p < 10^{-4}$) and MM ($p < 10^{-4}$), but not for V1 ($p = 0.48$) and A ($p = 0.03$). The difference in the test segment was significant for V1 ($p = 0.0002$), RSC ($p < 10^{-4}$), and MM ($p < 10^{-4}$), but not for A ($p = 0.053$). All p values were calculated by bootstrap for panels (a-d). The significance threshold was adjusted by Bonferroni correction with $\alpha = 0.05$ to account for 4 area-wise comparisons for panels (a-c), and 6 between-area comparisons for panel (d). V1: n = 1744 cells (84% of detected cells), RSC: n = 3865 cells (90 %), MM: n = 2310 cells (95%), A: n = 1105 cells (99%) for panels (a-f).

(b) Similar to Figure 4e, except for the first 0.5 s in the test segment. The difference between correct and error trials was significant for RSC ($p < 10^{-4}$), but not for V1 ($p = 0.08$), MM ($p = 0.17$), and A ($p = 0.16$).

(c) Similar to Figure 4f, except for the first 0.5 s in the test segment. The difference between correct and error trials was significant for V1 ($p < 10^{-4}$), RSC ($p < 10^{-4}$), and MM ($p < 10^{-4}$), but not for A ($p = 0.69$).

(d) Similar to Figure 4g, except for the first 0.5 s in the test segment. Difference in XOR information between correct and error trials in the test segment, calculated per cell and averaged across cells. Error bars indicate mean ± s.e.m. The difference between areas was significant between A and other three areas ($p < 10^{-4}$), but not between V1 and RSC ($p = 0.87$), V1 and MM ($p = 0.10$), RSC and MM ($p = 0.02$).

(e) Similar to Figure 4h, except for the first 0.5 s in the test segment. For each cell (circle), the sample cue information in the test segment and the test cue information in the test segment on correct trials (top/black) and error trials (bottom/magenta).

(f) Similar to Figure 4i, except for the first 0.5 s in the test segment. Distribution of cells from panel (e) in discrete polar angle bins for correct (black) and error (magenta) trials. Bins for $\theta < 0^\circ$ and $90^\circ < \theta$ show the fraction of cells that incorrectly encoded the cue identity (gray shading). Cells with noise-level information (magnitude $r < 0.01$) were not assigned angles but included in the total number of cells to calculate the fractions. The fraction of cells was significantly different between correct and error trials ($p < 0.002$) in the following bins; V1: $\theta < 0^\circ$, $90^\circ < \theta$; RSC: all bins except for $60^\circ \leq \theta < 90^\circ$; MM: $\theta < 0^\circ$, $60^\circ \leq \theta < 90^\circ$, $90^\circ < \theta$. The significance threshold was adjusted by Bonferroni correction with $\alpha = 0.05$ to account for 7 bin-wise comparisons. Error bars indicate s.e.m and are smaller than the data marker for some bins.

(g) Similar to Figure 4j, except for the first 0.5 s in the test segment. Top: Normalized mean activity of mixed selectivity cells for the four trial types on correct trials. Bottom: mean activity on error trials scaled by the normalization factors computed on correct trials. Nonlinearity Index of mixed selectivity cells ($15^\circ \leq \theta < 75^\circ$ in panel (e)) on correct trial was 0.57 ± 0.04 (mean ± s.e.m.) for V1 (n = 93 cells), 0.47 ± 0.02 for RSC (n = 183 cells), 0.46 ± 0.03 for MM (n = 65 cells). The mean activity for the preferred trial type was significantly lower on error trials than on correct trials in V1 (p = 0.0002), RSC ($p < 10^{-4}$), and MM ($p < 10^{-4}$).

(h) Similar to Figure 4k, except for the first 0.5 s in the test segment. XOR information for cells across angles (running average, window of 50 cells) for correct (black) and error (magenta) trials. The angle was defined on correct trials in panel (e). Shading indicates mean ± s.e.m. Cells with noise-level information (magnitude $r < 0.01$) were excluded. V1: n = 449 / 442 cells, RSC: n = 835 / 844 cells, MM: n = 329 / 331 cells were included for the analysis of correct / error trials.

(i) Similar to Figure 4l, except for the first 0.5 s in the test segment. Comparison of XOR information on correct (black) and error (magenta) trials, controlling for the sample cue information immediately before making decisions (last 0.35 s of the delay segment). Data points indicate individual trials, showing the sample cue information and XOR information averaged across simultaneously imaged cells in each trial. The running mean (window of 100 trials) is shown with shading indicating mean ± s.e.m. Gray bar at the bottom indicates bins of sample cue information in which XOR information was higher on correct trials than on error trials (p < 0.05, bootstrap). The correct-error trial difference was significantly larger in RSC compared to V1 for 0.01-0.03 logLR (2 bins) of the sample cue information. V1: n = 1476 correct / 307 error trials, RSC: n = 1420 correct / 265 error trials, MM: n = 1009 correct / 221 error trials.

Source data are provided as a Source Data file.

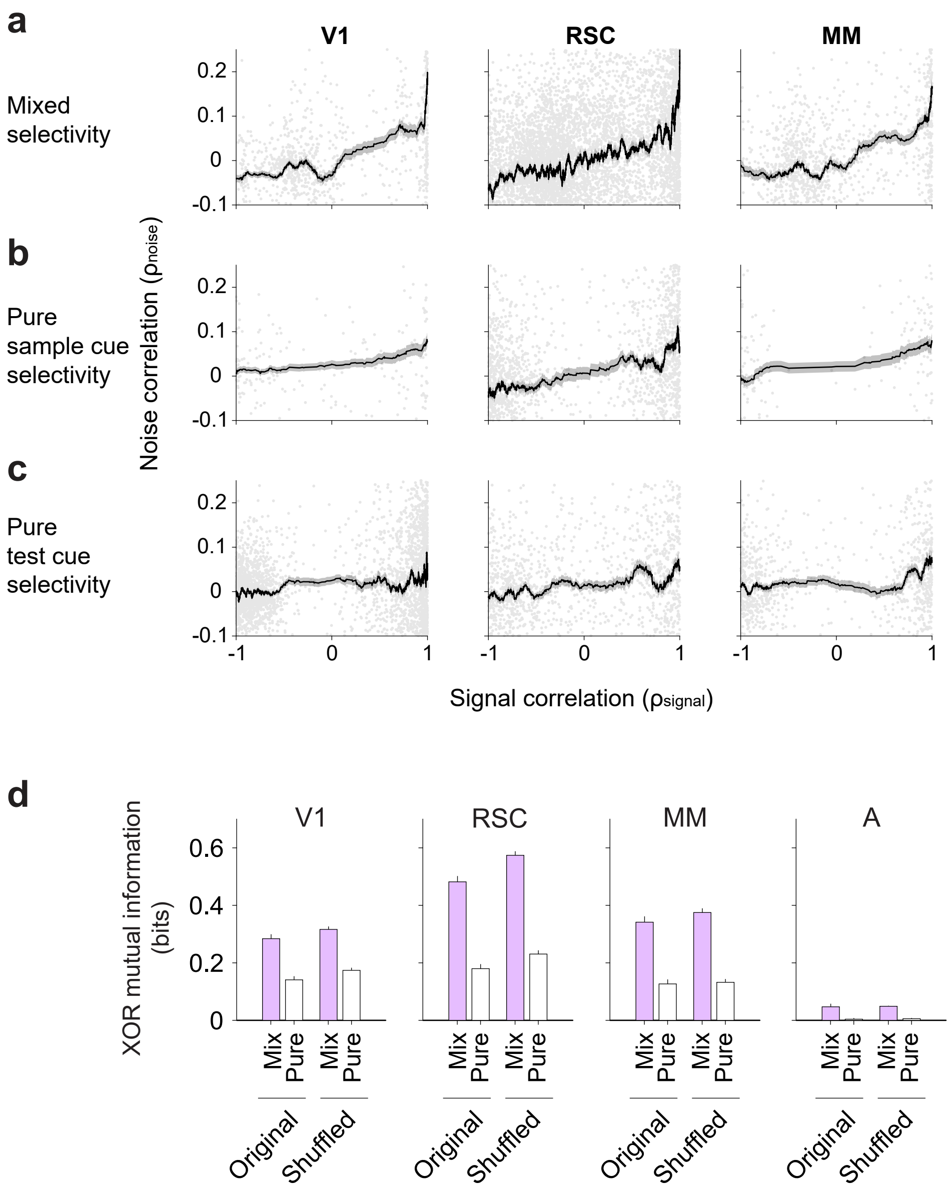

Supplementary Fig. 9 | Noise correlations in populations of mixed selectivity or pure selectivity cells

(a) Signal and noise correlations of neural activity for pairs of mixed selectivity cells (15˚≤ θ < 75˚ in Fig. 4h, correct trials) at the begging of the test segment (first 1 s). Gray points show Pearson's correlation coefficients for signal (mean activity for each of four trial type) and noise (residual activity around the mean). Black traces show the running mean ± s.e.m. (window size = 100 pairs). n = 1020 pairs in V1, 6807 pairs in RSC, 1177 pairs in MM.

(b) Similar to panel (a), except for pairs of pure sample cue selectivity cells (θ = 0˚ in Fig. 4i, correct trials). n = 202 pairs in V1, 1797 pairs in RSC, 182 pairs in MM.

(c) Similar to panel (a), except for pairs of pure test cue selectivity cells (θ = 90˚ in Fig. 4i, correct trials). n = 3362 pairs in V1, 1334 pairs in RSC, 958 pairs in MM.

(d) Decoding accuracy of XOR in the original data and data shuffled to disrupt noise correlations. Noise correlations were disrupted by shuffling trial identity within each of eight trial conditions (correct or error trials in the four trial types) for each cell. Error bars indicate mean ± s.e.m. V1: n = 11 sessions (1783 trials), RSC: n = 12 sessions (1685 trials), MM: n = 7 sessions (1230 trials), A: n = 5 sessions (822 trials). The difference in decoding accuracy between the mixed selectivity and pure selectivity population was not significantly different between the original data and trial-shuffled data in all four areas (V1: p = 0.66, RSC: p = 0.22, MM: p = 0.40, A: p = 0.43).

Source data are provided as a Source Data file.

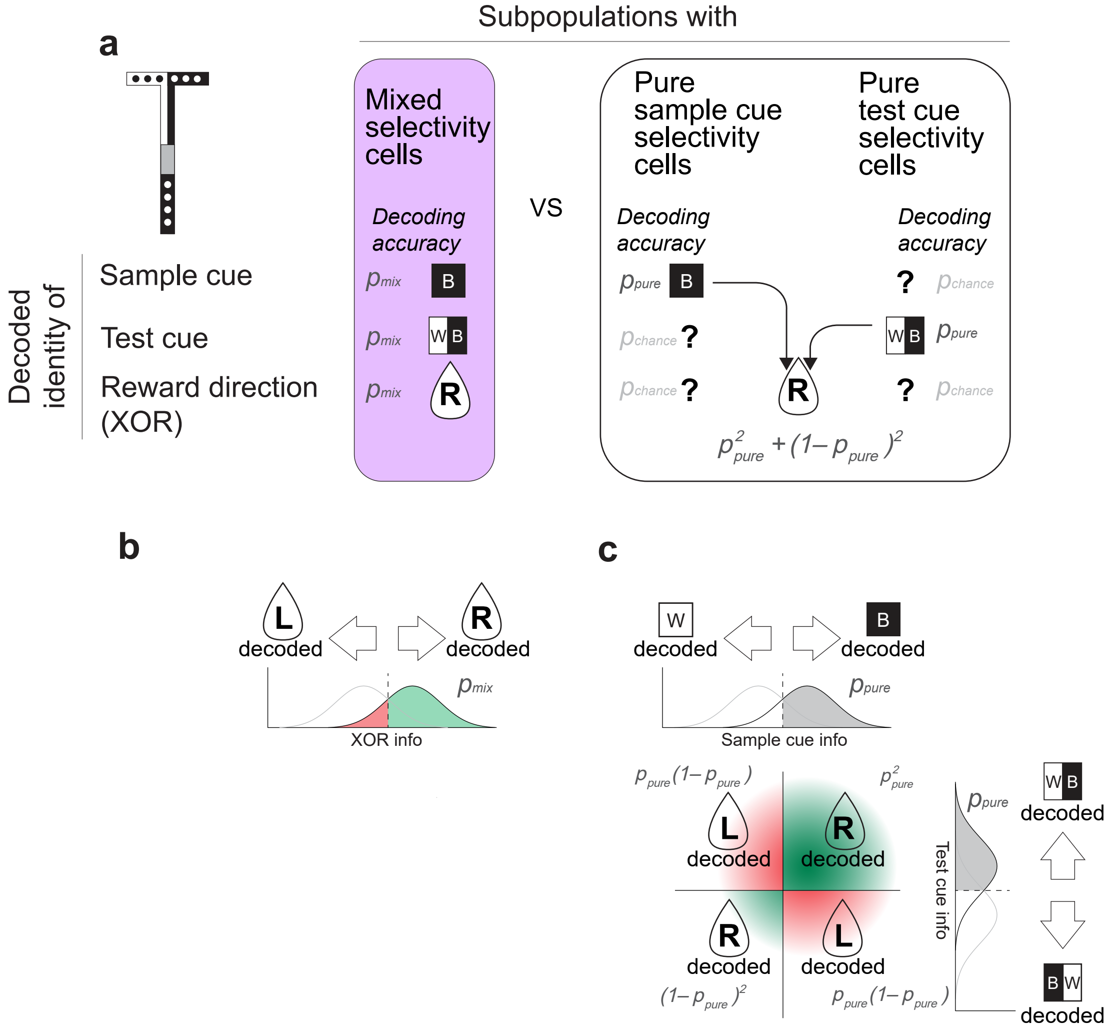

Supplementary Fig. 10 | Mathematical interpretation of the decoding accuracy from simulated mixed and pure selectivity populations

(a) Decoding accuracy for each task-related variable in mixed vs pure selectivity populations, shown similarly to Figure 6a.

(b) For a population of mixed selectivity cells, XOR can be linearly decoded by a single decision. The decoding accuracy is equal for each task-related variable (p_{mix}) because each cell is equally informative for the sample cue, test cue, and reward direction.

(c) For a population of pure selectivity cells, XOR can be decoded by dual decisions about the sample cue and test cue, as illustrated by the two decision boundaries for the sample cue and test cue (dashed lines). When the sample cue information and test cue information are plotted together in a 2D space, these two boundaries form nonlinear decision boundaries that separate right vs left (green vs red) decoding for the reward direction (XOR). When the decoding accuracy is equal for the sample cue and the test cue (p_{pure}), the decoding accuracy of XOR is given by $p_{pure}^2 + (1 - p_{pure})^2$, the sum of the probabilities that both cues are decoded correctly, p_{pure}^2, or incorrectly, $(1 - p_{pure})^2$ (due to the task design). The decoding accuracy for XOR is thus lower than that for the sample cue or test cue. See also Figure 7.

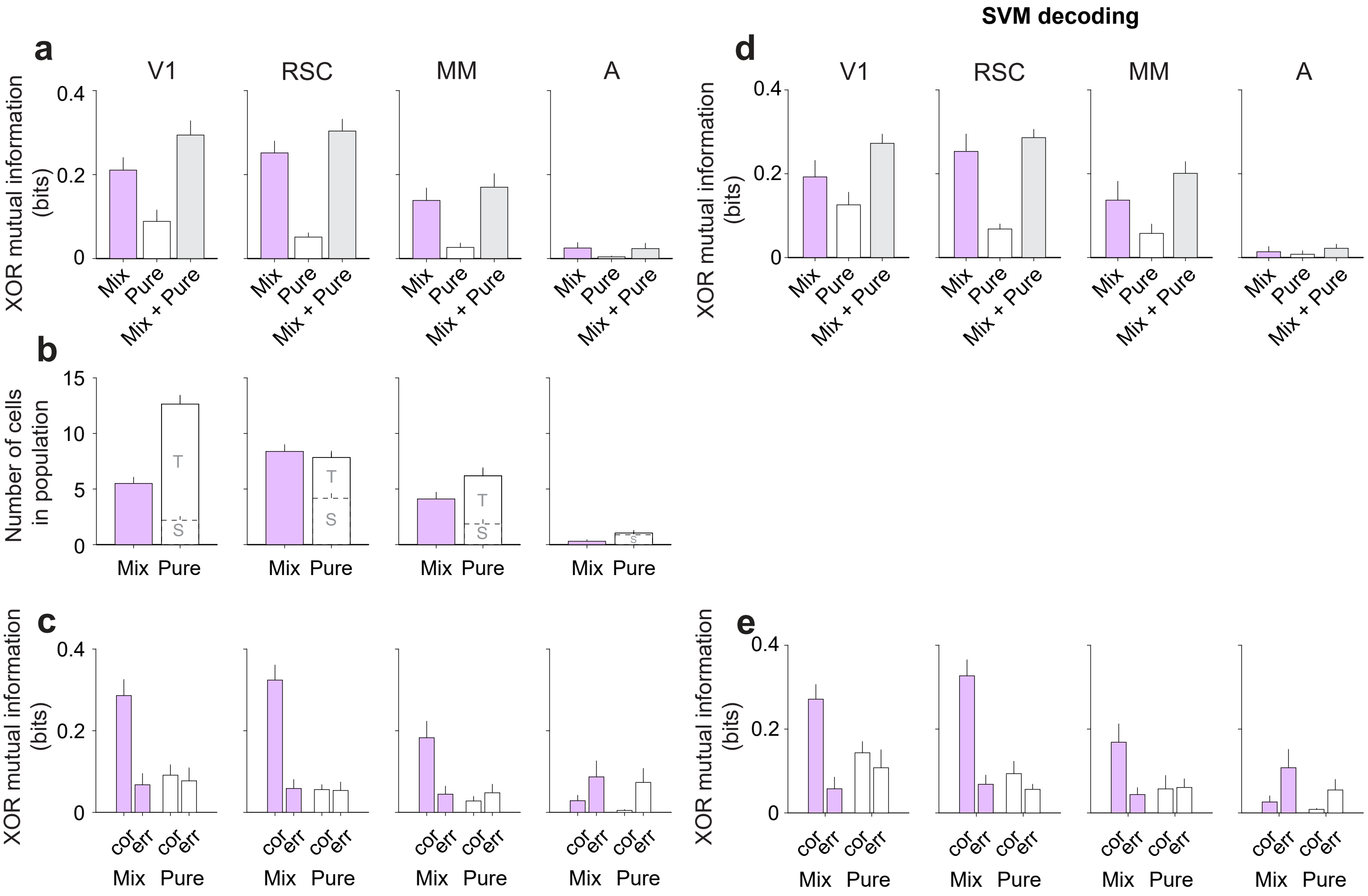

Supplementary Fig. 11 | XOR information in populations of mixed selectivity or pure selectivity cells, controlling for the population size

(a) Similar to Figure 6b, except for a population size of 100 cells. From the entire population imaged during each session, 100 cells were randomly sampled without replacement. The analyses included sessions with more than 100 detected cells. Mutual information between true and decoded XOR is shown for populations of mixed selectivity cells (15˚≤ θ < 75˚ in Fig. 4h, correct trials), pure selectivity cells (θ = 0˚ and θ = 90˚ in Fig. 4i, correct trials), and both types of cells combined (gray). V1: n = 8 sessions (1334 trials), RSC: n = 12 sessions (1685 trials), MM: n = 7 sessions (1230 trials), A: n = 5 sessions (822 trials). Error bars indicate mean ± s.e.m. across trials. Mutual information in mixed selectivity cells was significantly greater than that in pure selectivity cells in V1 (p=0.0016), RSC ($p < 10^{-4}$), MM (p=0.0002), but not in A (p = 0.11). All p values were calculated by random sampling of 100 cells in each session 10,000 times for panels (a-c), and the significance threshold was adjusted by Bonferroni correction with α = 0.05 to account for 4 area-wise comparisons for panels (a-c), and 6 between-area comparisons for panel (c).

(b) Similar to Figure 6c, except for a population size of 100 cells. Number of cells classified as mixed selective or pure selective (T = test cue selective; S = sample cue selective) per session is shown. Error bars indicate mean ± s.e.m. The number of cells was significantly larger for pure selectivity cells than for mixed selectivity cells in V1 ($p < 10^{-4}$) and A (p = 0.008), but not in RSC (p = 0.59) and MM (p = 0.034).

(c) Similar to Figure 6d, except for a population of 100 cells. Mutual information between true and decoded XOR on correct and error trials is shown. Error bars indicate mean ± s.e.m. V1: n = 8 sessions (1062 correct / 272 error trials), RSC: n = 12 sessions (1420 correct / 265 error trials), MM: n = 7 sessions (1009 correct / 221 error trials), A: n = 5 sessions (753 correct / 69 error trials). For mixed selectivity cell population, the difference between correct and error was significantly different from in V1 ($p < 10^{-4}$), RSC ($p < 10^{-4}$), MM (p = 0.0018), but not in A (p = 0.026). For pure selectivity cell population, the difference between correct and error was significantly different from zero in A (p = 0.012), but not in V1 (p = 0.66), RSC (p = 0.95), and MM (p = 0.33). The difference between correct and error in mixed selectivity cells was significantly larger than that for pure selectivity cells in V1 ($p < 10^{-4}$), RSC ($p < 10^{-4}$), and MM (p=0.001), but not in A (p = 0.84). The difference between correct and error in mixed selectivity cells was significantly larger in RSC than A ($p < 10^{-4}$), but the difference was not significant between RSC and V1 (p = 0.21) or MM (p = 0.02).

(d) Similar to panel (a), except with support vector machines with the radial basis function kernel (see Methods).

(e) Similar to panel (c), except with support vector machines with the radial basis function kernel.

Source data are provided as a Source Data file.

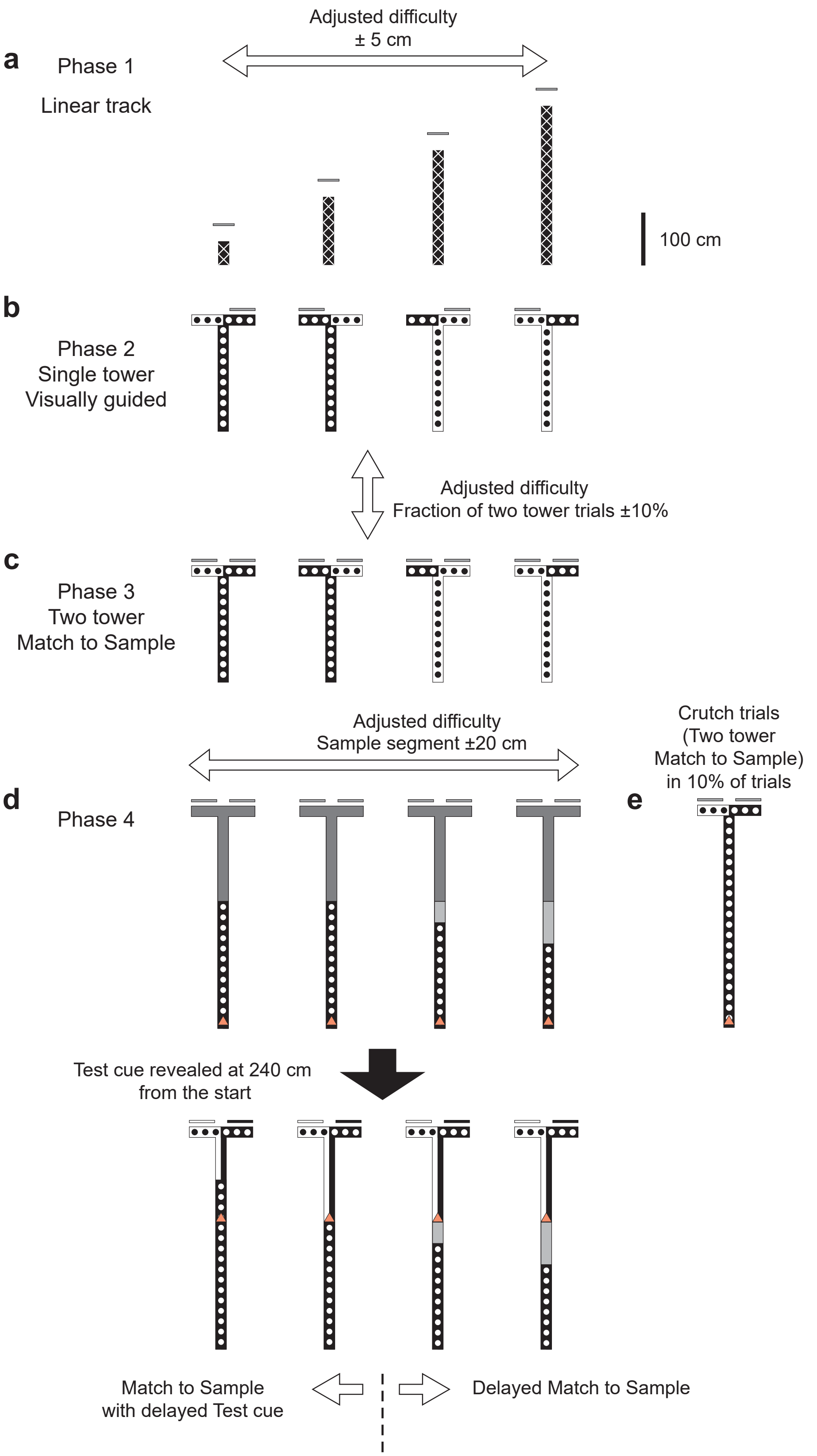

Supplementary Fig. 12 | Virtual reality maze for behavioral training

(a) Linear track in Phase 1. The maze was lengthened based on the behavioral performance (Methods).

(b) Single-tower maze in Phase 2. The T-stem was either black or white and T-arms were BW or WB, resembling the sample cue and test cue, respectively. The rewarded turn direction was determined by the combination of the T-stem color and T-arm color, but also indicated by the location of a single tower.

(c) Two-tower maze in Phase 3. The rewarded turn direction was determined by the combination of the T-stem color and T-arm color. Two-tower trials were interleaved with single-tower trials. The fraction of single-tower trials varied based on the behavioral performance (Methods).

(d) Delayed match-to-sample maze in Phase 4. Red triangle indicates the mouse's position in the maze. The test cue configuration, BW or WB, was revealed when the mouse reached a point 240 cm away from the maze start.

(e) Two-tower maze used in "crutch trials", which are randomly interleaved 10% of trials in Phase 4 to aid the performance of mice. The maze structure was similar to panel (c), except for the longer T stem.
