# Supplementary material for: A distributed and efficient population code of mixed selectivity neurons for flexible navigation decisions
Source: Nat Commun. 2023 Apr 14;14:2121. doi: 10.1038/s41467-023-37804-2 (PMC10102117; doi:10.1038/s41467-023-37804-2)
Supplement: Supplementary file 3 — Reporting Summary [file 41467_2023_37804_MOESM3_ESM.pdf]

nature portfolio

Corresponding author(s): Christopher D. Harvey

Last updated by author(s): Feb 17, 2023

Reporting Summary

Nature Portfolio wishes to improve the reproducibility of the work that we publish. This form provides structure for consistency and transparency in reporting. For further information on Nature Portfolio policies, see our Editorial Policies and the Editorial Policy Checklist.

Statistics

For all statistical analyses, confirm that the following items are present in the figure legend, table legend, main text, or Methods section.

n/a	Confirmed	
☐	☒	The exact sample size (*n*) for each experimental group/condition, given as a discrete number and unit of measurement
☐	☒	A statement on whether measurements were taken from distinct samples or whether the same sample was measured repeatedly
☐	☒	The statistical test(s) used AND whether they are one- or two-sided<br>*Only common tests should be described solely by name; describe more complex techniques in the Methods section.*
☐	☒	A description of all covariates tested
☐	☒	A description of any assumptions or corrections, such as tests of normality and adjustment for multiple comparisons
☐	☒	A full description of the statistical parameters including central tendency (e.g. means) or other basic estimates (e.g. regression coefficient) AND variation (e.g. standard deviation) or associated estimates of uncertainty (e.g. confidence intervals)
☐	☒	For null hypothesis testing, the test statistic (e.g. *F*, *t*, *r*) with confidence intervals, effect sizes, degrees of freedom and *P* value noted<br>*Give P values as exact values whenever suitable.*
☐	☒	For Bayesian analysis, information on the choice of priors and Markov chain Monte Carlo settings
☐	☒	For hierarchical and complex designs, identification of the appropriate level for tests and full reporting of outcomes
☐	☒	Estimates of effect sizes (e.g. Cohen's *d*, Pearson's *r*), indicating how they were calculated

Our web collection on statistics for biologists contains articles on many of the points above.

Software and code

Policy information about availability of computer code

Data collection

Imaging data were collected by Scanimage (Vidrio Technologies).

Behavioral data were collected by ViRMEn (Virtual Reality Mouse Engine) (v17-Jan-2015)
MATLAB code: https://pni.princeton.edu/pni-software-tools/virmen

Data analysis

Calcium imaging movie was motion corrected by custom code
MATLAB code: https://github.com/HarveyLab/Acquisition2P_class

Calcium fluorescence sources were detected by Suite2P.
MATLAB code: https://github.com/cortex-lab/Suite2P

Calcium imaging deconvolution was performed by OASIS
MATLAB code: https://github.com/zhoupc/OASIS_matlab

GLMs were fitted by glmnet (v2.0-16)
R code: https://github.com/cran/glmnet/releases/tag/2.0-16

Population decoding was performed by libsvm (v3.25)
MATLAB code: https://github.com/cjlin1/libsvm/releases/tag/v325

Other custom analysis codes were written in MATLAB.

For manuscripts utilizing custom algorithms or software that are central to the research but not yet described in published literature, software must be made available to editors and reviewers. We strongly encourage code deposition in a community repository (e.g. GitHub). See the Nature Portfolio guidelines for submitting code & software for further information.

Data

Policy information about availability of data

All manuscripts must include a data availability statement. This statement should provide the following information, where applicable:

- Accession codes, unique identifiers, or web links for publicly available datasets
- A description of any restrictions on data availability
- For clinical datasets or third party data, please ensure that the statement adheres to our policy

The data that support the findings of this study are available from the corresponding author upon reasonable request.

Allen Mouse Brain Common Coordinate Framework was used to show area borders in the dorsal cortex.
https://atlas.brain-map.org/

Human research participants

Policy information about studies involving human research participants and Sex and Gender in Research.

Reporting on sex and gender

Use the terms sex (biological attribute) and gender (shaped by social and cultural circumstances) carefully in order to avoid confusing both terms. Indicate if findings apply to only one sex or gender; describe whether sex and gender were considered in study design whether sex and/or gender was determined based on self-reporting or assigned and methods used. Provide in the source data disaggregated sex and gender data where this information has been collected, and consent has been obtained for sharing of individual-level data; provide overall numbers in this Reporting Summary. Please state if this information has not been collected. Report sex- and gender-based analyses where performed, justify reasons for lack of sex- and gender-based analysis.

Population characteristics

Describe the covariate-relevant population characteristics of the human research participants (e.g. age, genotypic information, past and current diagnosis and treatment categories). If you filled out the behavioural & social sciences study design questions and have nothing to add here, write "See above."

Recruitment

Describe how participants were recruited. Outline any potential self-selection bias or other biases that may be present and how these are likely to impact results.

Ethics oversight

Identify the organization(s) that approved the study protocol.

Note that full information on the approval of the study protocol must also be provided in the manuscript.

Field-specific reporting

Please select the one below that is the best fit for your research. If you are not sure, read the appropriate sections before making your selection.

☒ Life sciences ☐ Behavioural & social sciences ☐ Ecological, evolutionary & environmental sciences

For a reference copy of the document with all sections, see nature.com/documents/nr-reporting-summary-flat.pdf

Life sciences study design

All studies must disclose on these points even when the disclosure is negative.

Sample size	We did not predetermine the sample size before the experiments, but chose the sample size similar to our previous studies to attain statistical significance. We used 22 mice for this study. Of these, 10 mice were used for two-photon calcium imaging experiments, 12 mice were used for optogenetics inhibition experiments.
Data exclusions	Prior to any experiments, mice were excluded during training if they failed to reach steady high performance (greater than ~80% correct). Prior to any analyses, sessions with low behavioral performance (less than <~70%) were excluded. Prior to the analyses based on the GLM and quantification of information, cells were excluded if the model fit did not converge (typically due to temporally sparse activity).
Replication	We confirmed that the major results of the study were present in each individual animal by making a separate plot for each mouse.
Randomization	Mice were randomly allocated to experimental groups. In optogenetics inhibition experiments, inhibition sites and maze segments were chosen randomly by a computer program. Calcium imaging data were collected from multiple areas in each mouse and imaging sites were typically alternated between areas with different imaging depths.
Blinding	Blinding was not necessary because we investigated differences across cortical areas or cell types, as opposed to difference across individual mice (e.g., based on genotypes). Also, all analyses were performed objectively without human observation, so they did not require blinding.

Reporting for specific materials, systems and methods

We require information from authors about some types of materials, experimental systems and methods used in many studies. Here, indicate whether each material, system or method listed is relevant to your study. If you are not sure if a list item applies to your research, read the appropriate section before selecting a response.

Materials & experimental systems

n/a	Involved in the study	
☒	☐	Antibodies
☒	☐	Eukaryotic cell lines
☒	☐	Palaeontology and archaeology
☐	☒	Animals and other organisms
☒	☐	Clinical data
☒	☐	Dual use research of concern

Methods

n/a	Involved in the study	
☒	☐	ChIP-seq
☒	☐	Flow cytometry
☒	☐	MRI-based neuroimaging

Animals and other research organisms

Policy information about studies involving animals; ARRIVE guidelines recommended for reporting animal research, and Sex and Gender in Research

Laboratory animals	Mice were 8-16 weeks old at the start of behavioral training. They were housed in a group of 2-4 littermates in a 12 h: 12 h reverse light:dark cycle at an ambient temperature of 22 °C and ambient relative humidity of 50%. We used the following number of mice in this study.<br>8 male mice: C57BL/6J<br>12 male mice: VGAT-ChR2-EYFP<br>2 male mice: Thy1-GCaMP6s
Wild animals	The study did not involve wild animals
Reporting on sex	Sex was not considered as a biological variable. Only one sex was used for technical constraints related to differences in body size between sexes.
Field-collected samples	The study did not involve field-collected samples
Ethics oversight	All experimental procedures were approved by the Harvard Medical School Institutional Animal Care and Use Committee and were performed in compliance with the Guide for the Care and Use of Laboratory Animals.

Note that full information on the approval of the study protocol must also be provided in the manuscript.
